# Clinicopathological Significance of Estrogen Receptor *β* and Estrogen Synthesizing/Metabolizing Enzymes in Urothelial Carcinoma of Urinary Bladder

**DOI:** 10.3389/pore.2021.589649

**Published:** 2021-04-15

**Authors:** Naomi Sato, Kazue Ise, Shuko Hata, Shinichi Yamashita, Akihiro Ito, Hironobu Sasano, Yasuhiro Nakamura

**Affiliations:** ^1^ Division of Pathology, Sendai City Hospital, Sendai, Japan; ^2^ Division of Pathology, Faculty of Medicine, Tohoku Medical and Pharmaceutical University, Sendai, Japan; ^3^ Department of Pathology, Tohoku University Graduate School of Medicine, Sendai, Japan; ^4^ Department of Urology, Tohoku University Graduate School of Medicine, Sendai, Japan

**Keywords:** ERβ, urothelial bladder carcinoma, steroid metabolism, steroid sulfatase, aromatase, estrogen sulfotransferase 5

## Abstract

Sex-specific differences in the incidence of urinary bladder carcinomas are well known, and the possible involvement of sex steroids has been proposed. We previously reported the association of the loss of androgen receptors and androgen-producing enzymes with tumor progression of urinary bladder cancer patients. Clinically, the selective estrogen receptor modulators (SERMs) were reported to suppress the progression of these tumors but the status of estrogen receptors (ERs) has not been well studied in patients with bladder urinary cancer. Moreover, not only ERs but also estrogen-related enzymes, such as aromatase, steroid sulfatase (STS), and estrogen sulfotransferase (EST), have been reported in the biological/clinical behavior of various hormone-dependent carcinomas but not studied in urinary bladder carcinoma. Therefore, in this study, we immunolocalized ERs as well as estrogen metabolizing enzymes in urinary bladder carcinoma and performed immunoblotting and cell proliferation assays using the bladder urothelial carcinoma cell line, T24. The results revealed that the loss of STS and aromatase was significantly correlated with advanced stages of the carcinoma. *In vitro* studies also revealed that T24 cell proliferation rates were significantly ameliorated after treatment with estradiol or diarylpropionitrile (DPN). EST and aromatase were also significantly correlated with the nuclear grade of the carcinoma. The results of our present study, for the first time, demonstrated that biologically active estrogens that bind to ERs could suppress tumor progression and the inactive ones could promote its progression and the potential clinical utility of SERM treatment in selective patients with urinary bladder carcinoma.

## Introduction

Urinary bladder urothelial carcinoma is the seventh most common cancer worldwide, and its occurrence has recently increased. This carcinoma is also known to be three times more common in males than in females [1], but the reasons for this difference are unknown [2]. One proposed hypothesis is that androgens could play an essential role in regulating the onset of urinary bladder urothelial carcinoma [3, 4]. The results of previously reported epidemiologic studies indicated that postmenopausal women harbored an increased risk of developing bladder urothelial carcinoma in comparison to premenopausal women [5], suggesting the potential roles of androgens, as androgens become the more dominant steroids following menopause.

**FIGURE 1:**
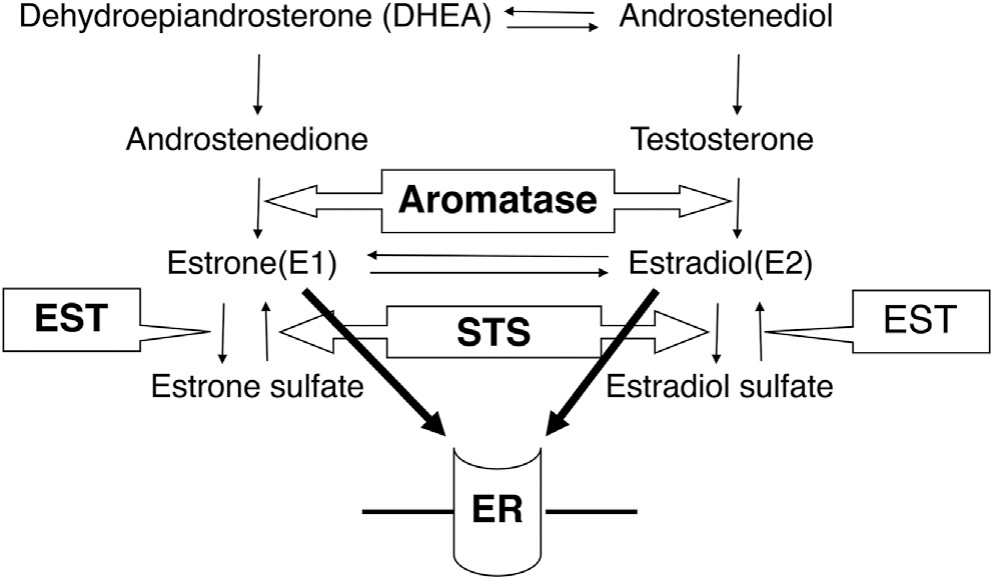
The correlation between steroid hormones and steroid metabolism enzymes. Aromatase converts androstenedione to estrone and testosterone to estradiol. STS inactivates estrone sulfate and estradiol sulfate, while estrone and estradiol are inactivated by EST. STS: steroid sulfatase, EST: estrogen sulfotransferase, ER: estrogen receptor.

In addition to androgens, estrogens are also well known to be involved in the development of several hormone-dependent carcinomas such as breast, endometrioid, and ovarian carcinomas [6]. Recently, not only estrogens themselves but also estrogen related enzymes have been proposed as a target of hormonal therapy, such as aromatase and aromatase inhibitor, which are used as a gold standard treatment for estrogen-dependent post-menopausal breast cancer patients [7]. Estrogens are also produced from circulating inactive steroids through steroid sulfatase (STS) and/or aromatase, while estrone or E1 is inactivated into estrone sulfate by estrogen sulfotransferase (EST) [6]. Figure 1 summarizes the correlation between steroid hormones and steroid metabolism enzymes. STS has been reported to be expressed in several tissues including the placenta and skin fibroblasts, breasts, and fallopian tubes [8] and has been detected in approximately 90% of breast carcinomas [9], 86% of endometrial carcinomas [10], several histological types of ovarian carcinomas [11], 85% of prostate carcinomas [12], and 61% of colon adenocarcinomas [13]. In addition, STS status has been reported to be correlated with poor survival rate in breast [14], endometrial [10], and ovarian cancer patients [15].

EST expression has also been reported in a wide range of adult and fetal tissues, including healthy urothelial cells and EST, which are considered to protect these tissues from excessive estrogenic effects [16]. ETS expression has been reported to be lower than STS expression in several malignant tissues, such as 40% of invasive breast carcinomas [17], 29% of endometrial carcinomas [10], 77% of prostate carcinomas [12], and 44% of colon carcinomas [13]. In contrast to STS, EST immunoreactivity has been reported to be inversely correlated with tumor size, lymph node status, risk of recurrence, and prognosis in the invasive breast carcinoma [17].

Estrogen receptors (ERs) are expressed in bladder urinary carcinoma cells [18]. In particular, the status of ERβ has been reported to be more abundant than that of ERα in urinary bladder carcinomas [19, 20]. The possible involvement of ERβ in bladder urothelial carcinoma development has been proposed, but its details are not known.

Therefore, in this study, for the first time, we examined the status of STS, EST, and aromatase in the bladder urothelial carcinoma tissue to understand the actions of estrogens further. We also conducted *in vitro* studies to substantiate the results obtained from immunohistochemical studies in clinical specimens of urinary bladder carcinoma.

## Materials and Methods

### Patients and Tissue Preparation

A total of 113 cases of primary urinary bladder urothelial carcinoma were retrieved from the surgical pathology files at Tohoku University Hospital (Sendai, Japan). These patients did not receive chemotherapy or hormonal therapy prior to surgery.

All enrolled patients signed the informed consents. The research protocols for this particular study were approved by the Ethics Committee at Tohoku University School of Medicine (2013-1-37).

### Immunohistochemistry

Immunohistochemistry was performed using the Histofine kit (Nichirei Biosciences, Tokyo, Japan). The antibodies used for immunohistochemistry of Ki-67, ERβ, STS, EST, and aromatase and the details of immunostaining are summarized in Table 1. The antigen-antibody complex was visualized using the 3.3’-diaminobenzidine solution and counterstained using hematoxylin. Ki-67 immunoreactivity was assessed by using the labeling index (LI in %) [21]. ERβ immunoreactivity was scored semiquantitatively, incorporating both the intensity and percentage of positive staining (H-score) [22]. STS, EST, and aromatase were examined in the cytoplasm of bladder urothelial carcinoma cells, and the cases harboring more than 10% positive carcinoma cells were tentatively considered positive in this study [12]. The stained slides were scored by two of the authors, and in case of any discordance, the case was observed under a multi-headed light microscope, and consensus was subsequently obtained.

**TABLE 1:** Antibodies used for immunostaining and immunoblotting.

Antibody	Host	Antigen retrival	Dilution	Application	Source
Ki-67	Mouse	Autoclave	100	IHC	Dako (Denmark)
ERβ	Mouse	Autoclave	1,000	IHC	Gene Tex (LA, United States)
ERβ	Rabbit	—	2,000	WB	Millipore (Germany)
STS	Mouse	—	100	IHC	Kyowa medex (Japan)
EST	Mouse	Microwave (pH9)	100	IHC	Sigma-aldrich (MO, United States)
Aromatase	Mouse	—	500	IHC	Dr Evans DB (ref. Sasano H. *J Steroid Biochem Mol Biol.* 2005)

IHC, immunohistochemistry; WB, western blotting; ER, estrogen receptor; STS, steroid sulfatase; EST, estrogen sulfotransferase.

### Cell Lines

The human bladder urothelial carcinoma cell line used in this study was the T24 cell line (American Type Cell Culture Collection (ATCC), Manassas, VA, United States). Cells were grown in McCoy’s 5 A medium (Gibco) containing 10% fetal bovine serum (FBS; Nichirei, Tokyo, Japan) and 1% penicillin-streptomycin.

### Immunoblotting ERβ Protein Expression Level in T24 Cells

The protein contents of T24 cells were extracted using M-PER (Pierce Biotechnology, Rockford, IL, United States) with Halt protease Inhibitor Cocktail (Pierce Biotechnology, Rockford, IL, United States) and protein concentration was determined using a Protein Assay Kit (Wako Pure Chemical Industries, Osaka, Japan). Proteins were resolved using SDS-PAGE (10% acrylamide gel) and transferred onto a PVDF membrane. After blocking with TBS-T (containing 0.05% Tween 20) and 5% non-fat dry skimmed milk for 1 h at room temperature (20°C), the membranes were incubated overnight at 4°C with the primary antibodies. The membranes were then washed and incubated for 1 h with horseradish peroxidase-conjugated goat anti-mouse/rabbit IgGs (GE Healthcare, Buckinghamshire, United Kingdom) at room temperature (20°C). Immunoreactivity was subsequently visualized through chemiluminescence (ECL Prime western blotting detection reagents: GE Healthcare).

### Cell Proliferation Assay

In the cell proliferation assay, T24 cells were treated with E2 or ERβ agonist DPN in a 96-well plate. For the steroid treatments, we incubated the cells in phenol red-free McCoy’s 5 A medium (HyClone) supplemented with dextran-coated charcoal-stripped FBS (fetal bovine serum) and 1% penicillin-streptomycin for 48 h. Cell proliferation was evaluated using the WST-8 colorimetric assay (Cell Counting Kit-8; Dojindo, Kumamoto, Japan). The status of cell viability was also evaluated as a ratio (%) compared with that of controls.

### Statistical Analysis

Statistical analyses were performed using R version 3.2.2 (The R Foundation for Statistical Computing Platform: x86_64-w64-mingw32/x64 (64-bit)). The correlation between various factors was evaluated using the Fisher’s precise test. *p* < 0.05 was considered statistically significant. Student’s *t*-test was used for the analysis of Ki-67 labeling index.

## Results

### Immunolocalization of Ki-67, ERβ, STS, EST, and Aromatase

**FIGURE 2:**
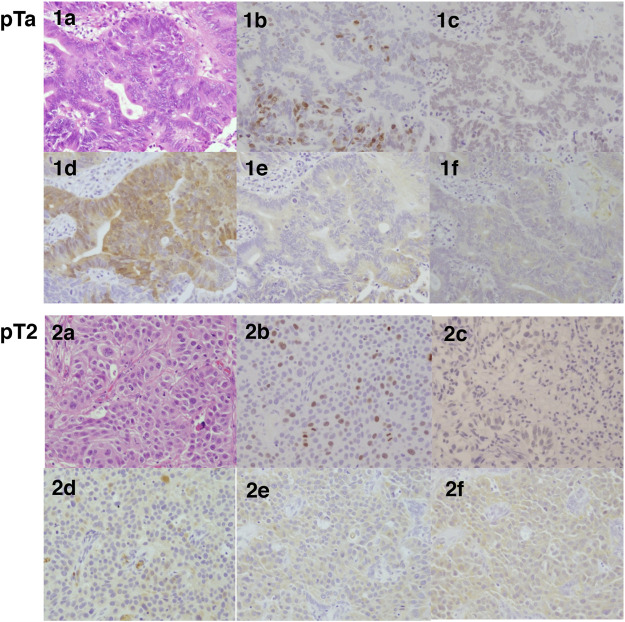
Representative hematoxylin-eosin and immunohistochemistry illustrations of pTa and pT2 histological sections in bladder urothelial carcinoma. Hematoxylin-eosin stained tissue sections **(a)**, Immunohistochemistry for Ki-67 **(b)**, ERβ **(c)**, EST **(d)**, STS **(e)**, and aromatase **(f)** in bladder urothelial carcinoma. 1a-1f, pTa; 2a-2f, pT2.

**FIGURE 3:**
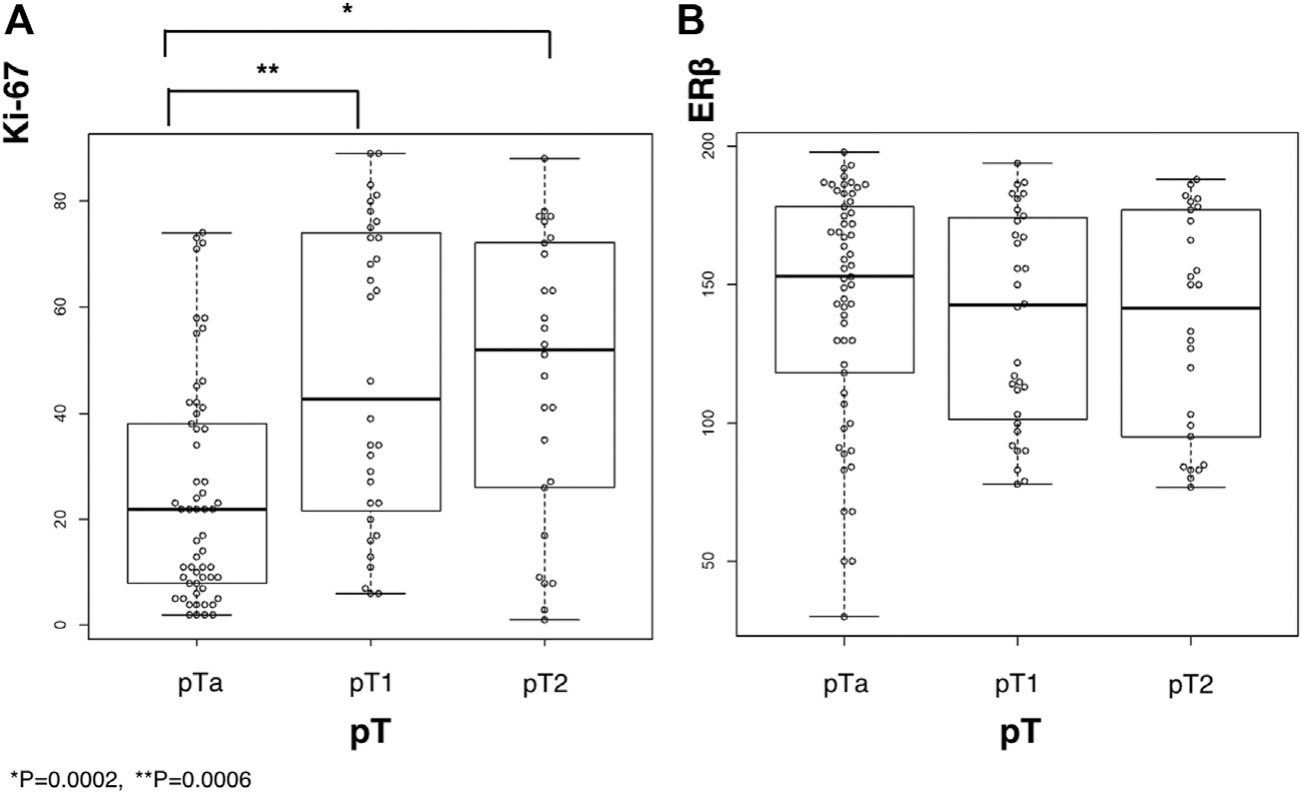
The results of immunoreactivity for ERβ and Ki-67 in bladder urothelial carcinoma along with their labeling index based on tumor stage. **(A)**, Ki-67 immunoreactivity; **(B)**, ERβ immunoreactivity. *p = 0.0002 and **p = 0.0006.

Immunoreactivity of Ki-67 and ERβ was predominantly detected in the nuclei of carcinoma cells (Figure 2), the average labeling index across the whole cohort and the tumor stage were summarized in Figure 3. The Ki-67 labeling index was significantly higher in pT2 than that in pT1 tumor grades (*p = 0.0002 and **p = 0.0006, respectively) (Figure 3A), but the status of ERβ immunoreactivity was not significantly correlated with the tumor grades (Figure 3B).

STS, EST, and aromatase were predominantly detected in the cytoplasm of carcinoma cells (Figure 2). STS and aromatase significantly decreased with the increasing tumor stages (Table 2, *p* = 0.0017 and *p* = 0.0430, respectively). EST and aromatase were significantly correlated with tumor nuclear grade (Table 3).

**TABLE 2:** Correlation between pT stage and immunoreactivities of STS, EST and aromatase.

		Positive	Negative	*p*-value
STS	pTa	43	14	0.0017^*^
	pT1	20	12	
	pT2	9	17	
EST	pTa	44	13	0.1900
	pT1	22	10	
	pT2	15	11	
Aromatase	pTa	15	42	0.0430^*^
	pT1	3	29	
	pT2	15	11	

* Indicates statistical significance.

Tumor depth is regulated by tumor-node-metastasis (TNM) classification based on the 8th edition of the TNM classification of malignant tumors (UICC, union for international cancer Control).STS, steroid sulfatase; EST, estrogen sulfotransferase.

**TABLE 3:** Correlation between nuclear grade and immunoreactivities of STS, EST and aromatase.

		Positive	Negative	*p*-value
STS	G1	2	3	0.1670
	G2	31	11	
	G3	38	30	
EST	G1	2	4	0.0490^*^
	G2	35	7	
	G3	38	42	
Aromatase	G1	0	5	0.0323^*^
	G2	51	39	
	G3	17	15	

* Indicates statistical significance.

Nuclear grades are determined by histopathological grading of tumor-node-metastasis (TNM) classification based on the 8th edition of the TNM classification of malignant tumors (UICC, union for international cancer Control).STS, steroid sulfatase; EST, estrogen sulfotransferase.

The status of Ki-67, ERβ, STS, EST, and aromatase was not significantly correlated with other clinicopathological parameters including age and sex of the cases examined.

### Effects of E2 or DPN Treatment on ERβ Protein Expression in T24 Cells

**FIGURE 4:**
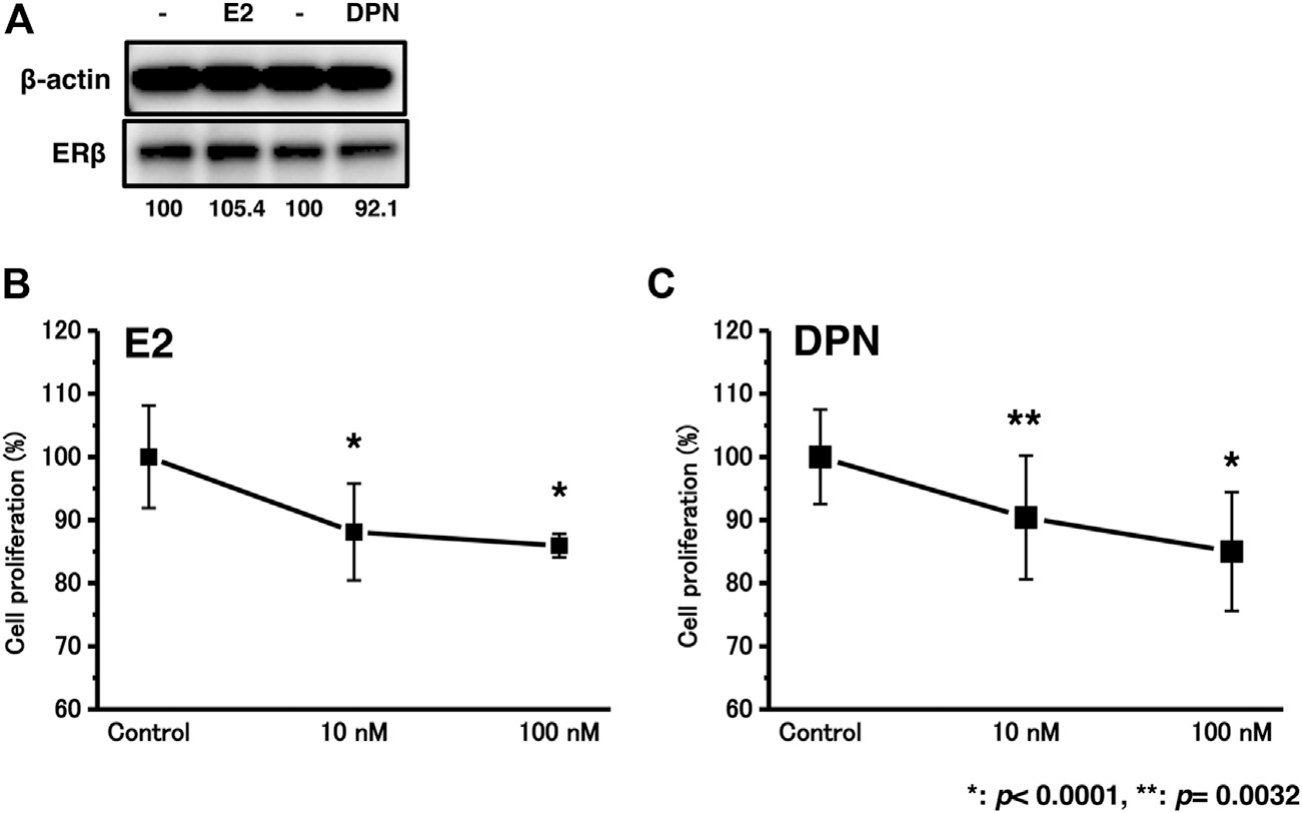
The effects of E2 or DPN treatment on bladder urothelial carcinoma cells. **(A)**, The effects of E2 or DPN on protein expression levels; **(B)**, Proliferation assay of T24 cells in the background of E2 treatment.; **(C)**, Proliferation assay of T24 cells in the background of DPN treatment. *p < 0.0001, **p = 0.0032.

The expression levels of the ERβ protein were not significantly influenced by E2 or DPN treatment (Figure 4A).

### Effects of E2 or DPN Treatment on Cell Proliferation in T24 Cells

We examined the effects of cell proliferation in T24 cells treated with 10–100 nM E2 or DPN. After treatment with E2 or DPN at 10–100 nM for 48 h, the proliferation rates of the T24 cells were significantly reduced (Figures 4B,C).

## Discussion

The possible correlation between urothelial bladder carcinomas and sex steroids has been long proposed, but the details are not known. The status of ER expression has been reported to be correlated with the progression or prognosis of urinary bladder carcinomas [19, 23–25], but others have not been able to replicate the findings and an inverse correlation has even been reported [26–29]. However, selective estrogen receptor modulators (SERMs) such as tamoxifen and raloxifen have been consistently reported to inhibit the proliferation and invasion of urinary bladder urothelial carcinoma [19, 20, 30–32]. SERMs are known to harbor high binding affinity for ERs, to function as ER agonists in some specific tissues such as bone, liver, and cardiovascular system, and also act as ER antagonists in the breasts and uterus [7].

Therefore, in this study, we first correlated the loss of STS and aromatase immunoreactivity in urinary bladder urothelial carcinomas with the increased tumor progression. In addition, the cell proliferation rates of T24 cells were significantly reduced following treatment with estradiol (E2) or diarylpropionitrile (DPN). In general, STS and aromatase have both been reported to be related to poor survival in many different carcinomas [10, 14, 15]. However, the results of our present study did demonstrate that STS and aromatase were both inversely related to the progression of urinary bladder urothelial carcinoma. These results, therefore, suggest that more active estrogen could be produced *in situ* in urothelial carcinoma, and could subsequently regulate the progression of the carcinoma. In addition, EST and aromatase were also significantly correlated with the nuclear grade of carcinoma, indicating that high-grade carcinoma could be associated with the local production/metabolism of estrogens. Previous study reported that ERβ expression and the non-cadherin switch were both accompanied by better recurrence-free survival of patients with urinary bladder urothelial carcinoma [28]. The mechanistic correlation between urinary bladder urothelial carcinoma and ERs is controversial; however the results of our present study are consistent with these findings.

A sex-specific difference (three times more common in men than in women) in the incidence of urinary bladder urothelial carcinoma is also known [1]. In addition, urinary bladder urothelial carcinoma accounts for 3% of all cancer deaths in men and 1.5% in women [2]. Between 1973 and 1999, there was a 33% decline in bladder cancer mortality; however, its reduction has only been detected in male patients [2]. This is because female patients tended to have more advanced disease at the time of diagnosis than men, resulting in higher mortality in women. For instance, 28% of male patients were diagnosed with stage III or IV disease, whereas 43% of the females were diagnosed with those stages [33]. The delay in diagnosis, cultural, biological, and anatomical reasons have been proposed as the reasons of sex discrepancy [2], but the results of our present study suggested that the different status of intratumoral estrogen metabolism could also account for this sex difference. The status of estrogen receptors was by no means correlated with any clinicopathological factors; however, STS and aromatase were inversely correlated with the progression of the disease. These results suggested that decreased levels of biologically active estrogens could result in increased tumor cell proliferation, progression, and adverse clinical outcome of urinary bladder urothelial carcinoma patients. Biologically active estrogens, which can bind the ERs, could ameliorate bladder urothelial carcinoma progression, and this is considered at least one of the reasons why SERMs could suppress the proliferation and invasion of urinary bladder urothelial carcinoma via ER-dependent induction [19, 20, 30–32]. The results of our present study could also account for the reasons why the expression of ERs alone was by no means correlated with the clinical behavior of urinary bladder urothelial carcinoma. However, to the best of our knowledge, the correlation among steroid receptors, sex, and age has not been reported yet. One of the possible reasons for this is that there are very few pre-menopausal female patients of urinary bladder urothelial carcinoma. Therefore, further investigations are required to clarify the sex differences of urinary bladder urothelial carcinoma.

## Conclusion

The results of our present study indicated that active estrogens could possibly ameliorate the progression of bladder urothelial carcinoma and that SERMs could be useful for the treatment of bladder urothelial carcinoma. Further investigations are required to elucidate the function of sex steroids in bladder urothelial carcinomas.

## Data Availability

The raw data supporting the conclusions of this article will be made available by the authors, without undue reservation.
